# Ten simple rules for developing visualization tools in genomics

**DOI:** 10.1371/journal.pcbi.1010622

**Published:** 2022-11-10

**Authors:** Eloi Durant, Mathieu Rouard, Eric W. Ganko, Cedric Muller, Alan M. Cleary, Andrew D. Farmer, Matthieu Conte, Francois Sabot

**Affiliations:** 1 DIADE, University of Montpellier, CIRAD, IRD, Montpellier, France; 2 Syngenta Seeds SAS, Saint-Sauveur, France; 3 Bioversity International, Parc Scientifique Agropolis II, Montpellier, France; 4 French Institute of Bioinformatics (IFB)—South Green Bioinformatics Platform, Bioversity, CIRAD, INRAE, IRD, Montpellier, France; 5 Seeds Research, Syngenta Crop Protection, LLC, Research Triangle Park, Durham, North Carolina, United States of America; 6 National Center for Genome Resources, Santa Fe, New Mexico, United States of America; Dassault Systemes BIOVIA, UNITED STATES

## Introduction

Visualization is key for expanding and communicating knowledge to both specialized and broad audiences—after all, “*a picture is worth a thousand words*,” right? That much has become clear during the Severe Acute Respiratory Syndrome Coronavirus 2 (SARS-CoV-2) outbreak when “*Flatten the curve*!” [1] turned into a catchier watchword than the usual “*Wash your hands*!,” by referring to the related graphics rather than the health discourses themselves. Coronavirus Disease 2019 (COVID-19) visualizations, good and bad [2], became omnipresent in the public debates, with interactive and dynamic visualization platforms like Nextstrain [3] gaining a lot of popularity.

We are now used to seeing graphs and charts in our everyday lives and creating them for scientific papers as part of research. Unfortunately, most of the time, classic visual representations are insufficient to effectively communicate data complexity, and as O’Donoghue puts it, “*often dedicated communication approaches need to be developed to address specific data challenges*, *especially when conveying complex or unfamiliar ideas*” [4]. Given the gap between creating static figures and building a visualization tool from the ground up, it can be easy to get lost in this nontrivial journey.

Our following 10 simple rules are dedicated to biologists and bioinformaticians who, while already being at the crossroads of many fields, want to venture further into the land of Data Visualization (“datavis” or “dataviz” for short). They combine tips and advice that we would have wanted when we first started our own journeys, gathered from our experiences in building genomic and/or datavis tools, and the time spent with related communities. Additionally, they address current challenges in computational biology and the needs of the community.

We aim these rules at bioinfo-to-datavis novices looking for guidelines, particularly regarding **genomics** visualization tools, but experienced practitioners may find it useful to see them gathered in one place too.

For better reading comfort, we organized the rules chronologically depending on when they would matter the most during a visualization tool’s life cycle, starting with the design (Rules 1 to 5) then development (Rules 5 to 8-ish) phases until it is shared with the rest of the world. Please note, however, that they are still relevant during the whole gestation and that creating a visualization tool is rarely a fully linear process—it does not even end after the initial release, as continued development and support is a cyclic process to be sustained over the years.

### Rule 1: Articulate the need for new visualization tools

For the past few decades, most genomics data have been visualized through genome browsers (JBrowse [5], Ensembl [6], IGV [7], etc.). With the advent of Next Generation Sequencing technologies, the number of draft or high-quality assembled reference genomes exploded, and the need to federate (gen)omic data across assemblies within and between species and to visualize them has become a major challenge.

All these data types and tools lead to critical questions, such as “***What am I trying to visualize*?**” and “***Why*?**”.

More specifically: What data do I have? Am I interested in structural variations? In epigenetic changes? At genome scale or at a defined locus? Are there gene annotations available? Can I get sequence alignments? Is there already a tool fulfilling all my expectations?…

Such questions must drive your quest for the appropriate datavis tool. A good first step would be to explore the related bibliography and tool reviews. Some reviews focus on the use cases [8] or layouts [9] of genomic visualization tools in general, while other reviews are specific to certain subjects, such as structural variations [10] or Hi-C data [11]. Your data and visualization needs may already be compatible with an existing tool or additional development of an existing tool could bring the feature you need. Getting a feature added to a tool can be done, for example, with GitHub by making a feature request or even adding the feature yourself with a pull request—consulting the dev team beforehand is usually a good idea, to be sure that what you propose is in line with their vision and community. Alternatively, some visualization tools allows the development of extensions or plugins, as exemplified by MetaPGN [12] which is built on top of a Cytoscape [13] plugin or the JBrowse plugin store (https://jbrowse.org/jb2/plugin_store).

If after all these steps nothing matches your needs, it then appears necessary to develop a new visualization tool. If so, your big challenge will be to **clearly identify the scope of your visualization tool** and to **keep your end goal in mind**.

### Rule 2: Involve others early on

There are many “others” that you could and often should work with when creating a datavis tool. Sedlmair and colleagues [14] identified 6 non-mutually exclusive collaborator roles of interest for design studies of such tools, with one of the main roles being the **front-line analysts**.

**Front-line analysts** are your end users, people who will use your tool and need it to complete certain tasks, and who should therefore be handled with particular care. It is crucial to engage them in discussion as early as possible since they are the ones who will (hopefully) adopt your tool. **Front-line analysts** can help you to properly define the tool tasks (i.e., both high- and low-level tasks that your tool should achieve) and provide test data as well as valuable feedback during both the design and development phases. There are many good ways to engage with your end users, including face-to-face interviews, surveys, design sprints, and even hands-on sessions that can often reveal edge cases and sometimes bugs that would have been hard to find on your own—beta-testing at its finest.

Moreover, there are fields dedicated to datavis: understanding how they are perceived by the human brain (visual perception and cognitive vision science), improving how they can be used with machines (Human Computer Interaction), and growing communities of **datavis designers**, full of people who could help build a visualization tool. They can be rather generalist, like the Data Visualization Society (https://www.datavisualizationsociety.org), or more dedicated to the visualization of life sciences data, such as the BioVis community (http://biovis.net), which regularly organizes conferences on related topics. Look around, there might be someone there willing to give you a hand!

### Rule 3: Think about visual scalability and resolution

If the history of genomics teaches us one thing, it is that what was once a huge dataset can be considered a toy set 5 years later [15]. The exponential growth of genomic datasets leaves us no choice but to consider the scalability of our tools’ design and the efficiency of their visual encodings (i.e., how well the chosen visual representations reflect the underlying data). Some designs might be good visual representations for small datasets, but scale poorly for larger datasets.

**Fig 1:**
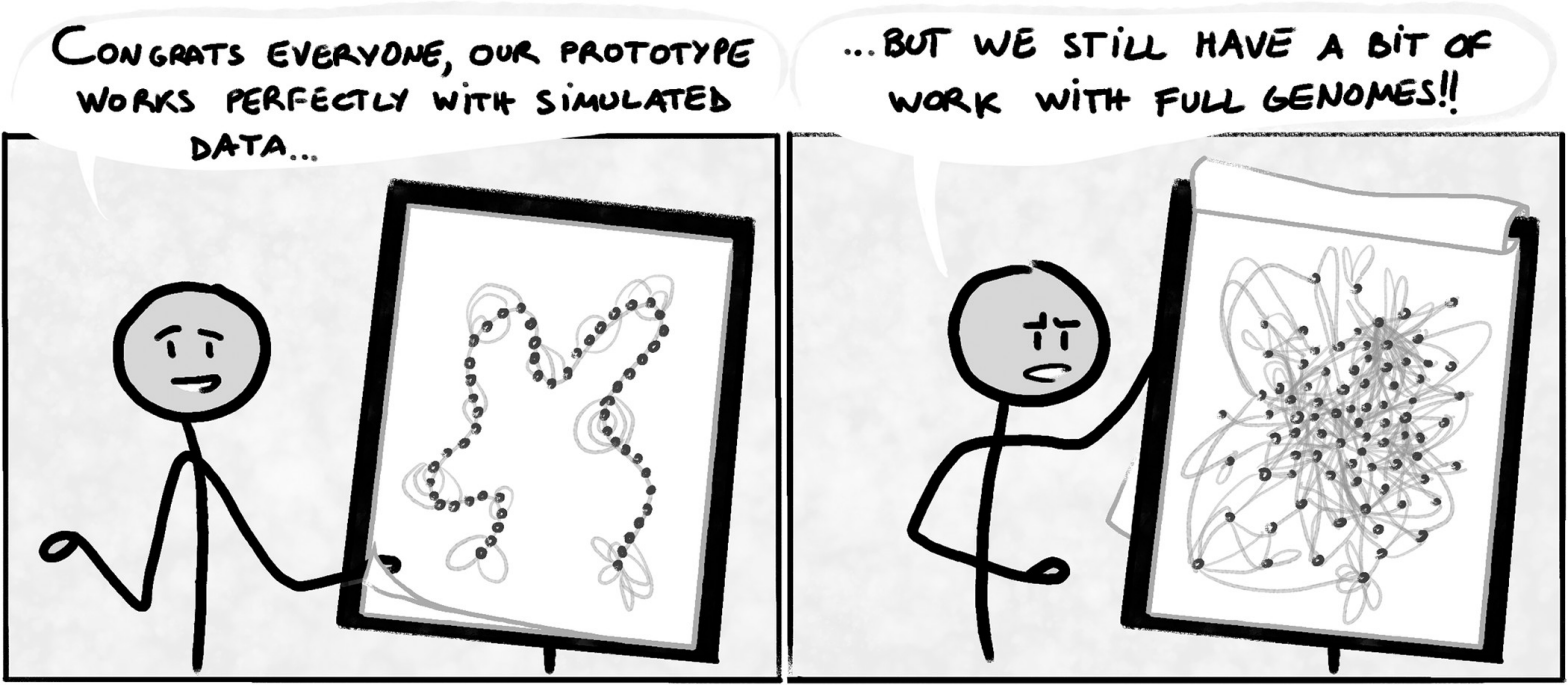
The readability of a visual encoding depends on the data provided.

For example, Venn diagrams are fine for up to 3 sets, but with more sets they become messy at best and a nightmare in worst case scenario. UpSet bypasses this issue by focusing on the set intersections and presenting them in an ordered “table,” but it still suffers a loss of readability with increasing sets (there is simply too much to see) and is not designed for visualizing hundreds of sets [16]. Similarly, networks and graphs do not adapt well to big datasets with many nodes and edges, resulting in the infamous “hairball effect” [17]. Your chosen visual encoding’s clarity can depend on your dataset, with different behaviors between simulated or real data of limited scope and larger, more complex data (Fig 1). **Your data will come with edge cases that you need to anticipate if you want your design to work properly at scale** [18]. Try varied configurations (good ol’ pen and paper are still useful to quickly draft multiple layouts), get familiar with your design’s limitations, and offer alternatives if you have the resources for it.

Genomic data has specificities and layers that you need to consider at different resolutions. Your organism(s) may have multiple chromosomes (linear or circular) to consider, heterozygosity or even polyploidy, and may present macro structural rearrangements at a chromosome level that will not interest you at the nucleotide sequence level. Along with the genomic sequences, you may also have access to additional data types, such as Hi-C, epigenomic signatures, or detections of transcription factor binding sites, all of which can blur the respective message in all-in-one visualizations. Instead, consider different visual representations for each data type and how they can complement each other through comparisons and interactions.

### Rule 4: Be creative, be bold

To develop a successful visualization application, you will have to find the right mix of technology, usability, and aesthetics.

Regarding technology, you can envision that scientists will soon have access to equipment similar to what Tom Cruise used in *Minority Report*, which will interact with virtual screens to perform multidimensional genomic data analysis. Virtual reality headsets and augmented reality devices offer a first, more affordable step in this unfamiliar 3D environment for datavis—some have already been put to use for GWAS [19] and visualization of 3D structure of chromatin [20] or genome graphs [21]. However, while technically feasible, such technologies are not popularized yet and that choice would likely reduce the number of end users. Some tools compromise by projecting a 3D space onto a 2D computer screen, like Graphia [22] that uses perspective views and shading to simulate depth perception. There are plenty of technology options available and you will need to keep up to date, but make sure that your audience can effectively and correctly use them!

As for aesthetic and artistic ways to present data, it is partly subjective. Something nice and eye-pleasing to one person may not be appreciated and understood the same way by another person. However, there is a good amount of literature providing advice for efficient datavis design [23–25]. One example is to pay special attention to how you use colors [26]. Make your tool more accessible by accommodating visually impaired people [27], who make up more than 3% of the global population [28], and consider providing a way to let users customize their visualizations with their own color patterns.

Overall, **be creative, do not be afraid to create designs that will not make it to the final cut**, and test different graphical strategies following user experience (UX) design processes [29]. Finally, even though too much novelty could be a barrier to adoption, a smart and beautiful design will encourage your users to engage more by offering them visual clarity of their data. If art is really your thing, though, you could totally find a place among the “*Xenographics*” (https://xeno.graphics) or create your own science-inspired pieces in your free time (check out Martin Krzywinski’s π-art gallery: http://mkweb.bcgsc.ca/pi/art)!

### Rule 5: Make data complexity intelligible

**Fig 2:**
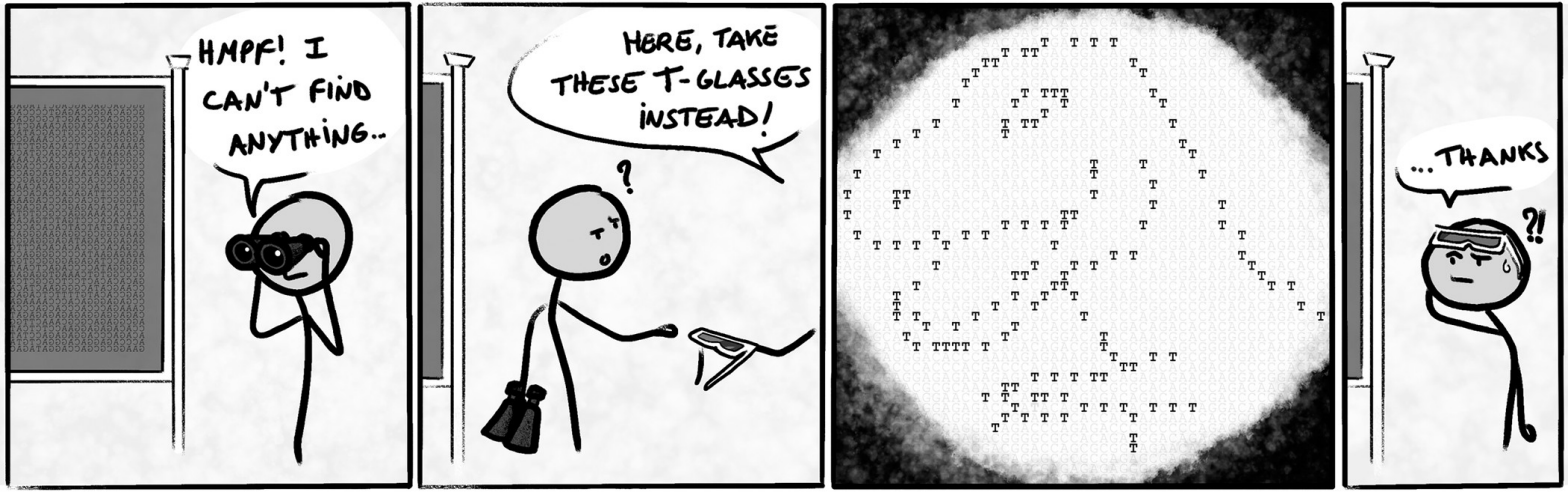
A good datavis makes pattern identification easier (feat. the Datasaurus).

A familiar way of making complex data accessible is to compute derived measures and statistics or to apply dimension reduction techniques. Still, visualization is recommended to detect patterns that would not be found with these means alone, as illustrated in Anscombe’s quartet [30], and the more recent Datasaurus Dozen [31]—based on Alberto Cairo’s eponymous dataset—which inspired the Fig 2. Unfortunately, the human mind cannot make sense of everything that it perceives at once, and our attention is instead focused on fragments of what we see.

In practice, our understanding of graphical representations comes in part from the immediate identification of salient elements that visually “pop out” (i.e., *preattentive processing* [32]), but it mainly comes from active exploration [33]. Therefore, a key component of datavis is to make exploration easier and to reduce the workload for a user’s brain starting with careful design as to relieve their working memory [24].

A universally praised rule of thumb to this end is Shneiderman’s mantra: “**O*verview first*, *zoom and filter*, *then details-on-demand***” [34]. This approach encourages visualization developers to make clever use of the main advantage of visualization tools over visual representations: interactivity. With such a divide and conquer approach it becomes easier to make sense of the displayed visual information as a user. For example, adding details with dynamic tooltips or including dedicated linked views enables users to build a deep understanding of the data provided without hiding the overall patterns.

### Rule 6: Let your inner nerd shine if needed

Technology matters, but it is not an end in itself. If you can design and produce an efficient tool using reliable methods instead of the latest and trendiest ones, there is no reason not to. Using the latest framework or published method can be interesting when they add significant advantages, but they may come with stability issues and will not always be the safest option for a robust and long-term tool (as an example, we can think of the infatuation with Objective-C in the mid-2000s leading to the creation of the BioCocoa framework (http://bioinformatics.org/biococoa), without any evolution since 2011). Moreover, accumulating niche technologies and methods would make it difficult to find someone with the exact skillset needed to maintain or help develop your tool; consider the consequences of that choice carefully.

On the other hand, it is also important to recognize that software technologies evolve quickly, and once-common approaches to user interface development may lose favor or become inviable (consider applets and other rich-client alternatives to browser-based development or the genomic visualization tool Gobe [35] that unfortunately came out after the first signs of Adobe Flash’s downfall). Sometimes a well-conceived and popular tool may need to be completely overhauled in order to remain relevant and usable. This is what happened with genome browsers whose implementations transitioned from HTML image-maps generated server-side to client-side JavaScript frameworks.

You will need to use relevant technologies for your tool: using LaTeX to produce pangenomics visualizations is fun but needlessly complicated [36] and there are better, dedicated technologies out there. Common tools in datavis differ from common tools in bioinformatics in general, and you will have to try and get accustomed to unfamiliar technologies and concepts. For web-based visualization, you should first familiarize yourself with the differences between Scalable Vector Graphics (**SVG**, geometrically defined images) versus **HTML5 Canvas** (dynamic pixel-defined images) elements. A must-know library for working with visualization is **D3.js**, which enables the manipulation of (graphic) elements on a webpage. Finally, if your goal is to display numerous elements at a time (say thousands or millions), you should consider working with **WebGL** (an API using the graphic card for faster display)—check out the Three.js library or the GenomeSpy [37] or Gosling [38] visualization grammars if interested.

### Rule 7: Benchmark with diverse datasets

While it is important to have a well-thought-out design, you cannot ignore performance, as a smooth user experience is key for your tool’s adoption [39]. Benchmarking with different datasets is critical for your visualization tool, and each type has its own use.

- **Small simulated** datasets can and should be used during development, as they are better suited for fast iteration while debugging. Make sure that what you see is faithful to the data you have. Handcrafted files can be useful to make extra sure that the resulting visuals match your expectations.
- **Big simulated** datasets are meant to be used for stress tests, to assess scalability and performance: how much data can you feed to your tool until it breaks? Until the tool starts slowing down too noticeably? Performance is an important aspect of your tool that should be determined, at least to let users know what to expect if they want to use their data with your tool.
- **Real** data, finally, to make sure that your tool is working properly with real-life data. Look out for unexpected behaviors and edge cases related to your design or data format loopholes that may hinder the user experience.

With the decrease of sequencing costs, it is quite frequent to have huge projects with hundreds if not thousands of samples: humans, *Arabidopsis*, mosquitoes, rice, bacteria, viruses, and so on. A modern tool would be expected to manage hundreds of datasets, with as few lags as possible and no memory crashes. Keep in mind that “hundreds of genomes” may not correspond to the same size depending on the studied organisms: 1,000 HIV genomes represent approximately 9.2 Mb, which is less than 0.14% the size of a single human genome. You should therefore consider performance through 2 axes: count capacity (number of genomes or measures that can be added) and size capacity (efficient and maximum file sizes that can be used). You may also need to consider characteristics such as the number of chromosomes (or scaffolds) within a genome as well as the distribution of sizes among these elements (e.g., an abnormally large chromosome in an otherwise “normally sized” genome could break the assumptions of your data structures).

If performance becomes an issue, compiled code and optimized graphics libraries could be a good alternative to scripting languages (e.g., JavaScript, python) to generate the display of large amounts of data.

### Rule 8: Be aware of the genomic tool ecosystem and promote interoperability

Your application will likely need to work among an existing ecosystem of databases and tools that vary by community, even when developed as a stand-alone instance. To lower your tool’s barriers to adoption, **pay special attention to implementation, distribution, documentation, and deployment**.

Regarding implementation, your tool should be as light as possible, OS agnostic [40], and if possible, run client-side. Implementing a standard Genomics API [41] to consume input datasets will also enable seamless integration and better interoperability with other databases and applications. Moreover, some users may want to include your visualizations in automated workflows, so the ability to get output via a command-line and companion scripts can be a nice additional feature to provide.

For distribution, consider multiple options—a Docker or Singularity containers [42,43], or the Conda [44]/Bioconda [45] environment management systems can make complex setups much easier to install, and providing users with the details to install with dependencies on other systems is also valuable. Web applications (e.g., those built with JavaScript) are great for avoiding multiple setup issues as they can work with any web browser.

When time and resources allow, making a popular tool available on several platforms and programming languages is a good option for sustainability. JBrowse is a good illustration since it is available in both web and desktop versions [5], can be embedded in large genome portals as a Drupal module [46], has an R markdown and R Shiny compliant version (JBrowseR [47]), and exists as a Jupyter package [48].

Hosting an example of your software on a website can be useful for trial purposes but can be difficult to maintain over time. Always seek to make it easy for potential users to try your tool on their systems—include clear instructions and a straightforward way to download and run the tool. Good trial data helps users understand the tool and is also a good test to make sure the tool is running properly following installation. Moreover, data formats in bioinformatics can be tricky [49] so clearly document the formats your tool needs and provide examples.

### Rule 9: Keep up to date with related work

Genomics has been quickly evolving in the past years and shows no sign of slowing down in the future. Envisioned future developments and challenges are linked to more diverse and applied omics approaches (especially in health), data ethics and security, reference-free studies and others, all with ever more data and a growing need for integrated solutions [50].

With fast evolving needs and many people working on these subjects, it can be easy to be the needle in a haystack and be forgotten in favor of another tool: As of May 30, 2022, there are 434 visualization tools listed in the *awesome-genome-visualization* list [51] and 151 research articles (already 13 out in 2022 and counting) found via Europe PMC whose listed keywords matched “*visualization*” and “*genomics*.” Make sure you keep up to date with data and technologies in genomics so that your ideas and tools stay fresh and relevant. Do not let your focus wander off too much either: A polished and thoroughly executed idea is better than a hybrid monstrosity of hastily (re)defined goals.

Moreover, you should try to **ride the wave rather than fight against it**: Make your work known and alive (in conferences, articles, Twitter…) and people will start noticing and using it. After all, you could very well make the new state-of-the-art tool!

### Rule 10: Grow and support your user community

Gaining users is the final key to a great visualization tool. To do that, you need to communicate your tool to prospective users as well as those who are actively working with it. **Communication can take time, but it is rewarding**—not only to ensure that people are using your tool, but also to gather ideas for improvements.

User documentation is a simple starting point—make sure you have an up-to-date README or manual for your software, including multiple examples on how to actually use your software. GitHub is an easy way to centralize this information and engage with those that have downloaded your project and are attempting to use it, especially through the “Discussions” and “Issues” features. Projects with no activity or updates are a warning sign to potential users, whereas those with activity are more enticing. Plus, you can centralize answers to frequent questions and, if you have an active user base, GitHub Discussions now supports polls, making it a convenient way to get input on new ideas. Furthermore, as reproducible research and FAIR-sharing principles are applicable to software products [52,53], it may encourage users to cite a released version of your tool by using tools like Zenodo to issue persistent Digital Object Identifiers (DOI) against your releases.

When presenting at a conference, try to end with 1 to 2 questions about new feature requests or biggest issues, along with contact details. You could create dedicated groups on online platforms like meet-up or organize training sessions towards federating a user community. Also consider setting up a google search notifications around the name of your tool—you might find mentions on Biostars, publications, and other sites you were not expecting.

Finally, do not forget to use social media to cast a wider net by advertising releases of the tool as well as occasional examples of its use [54].

## Conclusions

Our 10 simple rules for developing genomic visualization tools will not replace the experience gained by trial-and-error but will hopefully make the development process less painful for future bio-datavis practitioners and other datavis enthusiasts. Moreover, these rules merely cover the tip of the iceberg, and we strongly encourage our readers to take a look at the references included in this paper to further improve their understanding. Other interesting matters that did not make it through the introductory cut include visual design validation [55,56], inclusion and accessibility matters [57,58], and visual representation of uncertainty [59,60], which are subfields on their own.

Most of the elements presented here would also be applicable to non-genomic datavis tools; what may be more specific to genomics here is that the datasets are increasing at fast pace [15], with a target audience of scientists that have specialized questions covering a wide range of data types and scales. Depending on your goal (see Rule 1), you may have to create a multi usage tool that could work for pre-established analysis workflows and exploratory usages as well as for capturing printable versions of your visual representations at a given state.

These static snapshots could be used for publication, as you should also considering publishing papers about your tool and the design choices behind it (datavis-oriented columns are gaining in popularity, for example, with the datavis section in *Frontiers in Bioinformatics*).

To wrap up these 10 rules, here is a summary of what we deem most important, our take-home message for those who did not have time to read the article in detail:

- Going blindly into building a visualization tool is a bad idea; take time to learn about datavis principles first (we highly recommend Nature Methods’ “Points of View” column [61]) and to carefully consider your tool’s visual representations and interface designs.
- Genomics comes with multiple challenges, due in part to its diversity and scale of data. Make sure you have correctly identified the tasks your tool should complete and consider implementing interactions between different visual representations.
- Interact with your community at all times: during the design phase to correctly assess your end goal; during development to use real data and make sure you are going in the right direction; and on an ongoing basis once your tool is available for all to enjoy, to prevent it from ending in the “*oubliettes de l’histoire*.”

## Supporting information

S1 File Extraction processes and data used in Rule 9. Text file including the extraction method and request string used to count tools from the awesome genome visualization list and publications related to genomic visualization from PMC.
(TXT)
